# Practical and organisational problems in the testing of clinical laboratory instruments

**DOI:** 10.1155/S1463924678000097

**Published:** 1978

**Authors:** L. B. Roberts

**Affiliations:** Biochemistry Department Gartnavel General Hospital Glasgow GI2 0 YN UK


**Table 1 Some typical results obtained using the automatic solid liquid extraction module.**

| Parameter Determined | Matrix | Theoretical Value | Experimental Value | Standard Deviation | Number of samples |
|---|---|---|---|---|---|
| Titratable acid | Resin | 160mVal | 160.7mVal | 0.30% | 18 |
| Vitamin C (Ascorbic acid) | Capsule | 1000mg | 1001.2mg | 1.07% | 8 |
| Sulfonamide | Tablet | 500mg | 501.5mg | 0.41% | 6 |
| Simazine | Powder | 80% | 80.62% | 0.43% | 8 |
| Atrazine | Powder | 80% | 80.26% | 0.16% | 9 |

capsules, dry ampules, suppositories, creams, ointments and pastes using a variety of solvents such as water, aqueous dilute acids and bases, alcohol, acetone, chlorinated solvents and petroleum ether. The final measurements were carried out by direct photometry in either the UV or visible region, by indirect photometry or by a variety of titration methods, for example 0.1N solutions of HCl, NaOH, $NO_2$, $I_2$, Fe (III), Ce(IV), Ag(I), $HCO_4$, in glacial acetic acid and with TBAH. Some typical results are shown in Table 1.

Reproducibility of the automatic methods is equal or better than the equivalent manual procedure, the actual determined content of the analyte is in general equal to the manual result but in a few cases is slightly greater. A proven manual method can by simply and quickly translated into an automatic regime and results obtained within an hour. A laboratory technician can become proficient with the device with only one day's training. These features are a great advantage.

The extractor has a sample throughput of about 70 different analyses in an eight hour working day, extension into the silent hours will double this throughput. Where the analytical problem relates simply to checking sample uniformity a further doubling of sample throughput, is possible because some washing procedures can be omitted.

### Discussion

The design and construction of a highly reliable solvent extractor capable of precise analysis was made possible by close co-operation between the instrument company and a team of analysts working for a chemical manufacturer. The applicability of the device in routine analysis has been fully evaluated over an extended period of evaluation. These evaluations show that it is suitable for many applications and materials. The results obtained show that the inherent improved control over manual procedures produces increased precision of analysis.

REFERENCES

[1] C. R. Rehm, T. Urbanyi & T. J. Slone: A Versatile Automated System for the Spectrophotometric Analysis of Single Tablets. *Annals New York Acad. Sci* **153**, 640-654 (1968).

[2] D. G. Rohrbaugh & J. Ramirez-Munoz: Analytical Applications of an Automatic Material Analyzer, *Analytica Chimica Acta,* **71**, 311-320 (1974).

[3] P. Grafstein & R. Goldberg: The SOLIDPREP Sampler II and Automated Wet Chemical Analysis of Solid Samples, *Technicon International Congress 1972, Advances in Automated Analysis,* **9**, 53-59.

[4] V. Reicher: *Technicon Bibliography,* Technicon International 1973, Geneva, Switzerland.

[5] R. W. Arndt & R. Werder: Automation in Wet Chemical Analysis, Z. anal. Chemie **287**, 15-18 (1977).

[6] N. L. Alport: Automated Instruments for Clinical Chemistry, *Clin. Chem.* **15**, 1198 (1969).

[7] N. G. Anderson: Analytical Techniques for Cell Fractions, *Analytical Biochemistry* **31**, 272-278 (1969).

[8] P. V. Frueh, L. Meier, H. Rutishauser & O. Siroky: A Microcomputer-controlled Titrator for Automated Individual Analysis, *Anal. Chim. Acta* **95**, 77-106 (1977).

### Practical and organisational problems in the testing of clinical laboratory instruments

**L. B. Roberts**

*Biochemistry Department, Gartnavel General Hospital, Glasgow, G12 0YN, UK.*

**The** expenditure on complex instruments for clinical laboratories has increased over the last fifteen years both absolutely and relatively. The relative increase is, of course, conditioned by the rate of inflation over that period but for similar instruments, e.g. a pH meter, the cost of hardware has probably decreased when measured at both ends of a fifteen year time span. Absolute increases are due to the use of better instruments, in terms of design, reliability and function. Additionally the greater use of automatic instruments has also added to the absolute costs. A good example here is the move from single channel to multichannel analysers which although the capital sum of an equivalent number of single channel instruments is probably greater than that of the multichannel instrument, the necessity to expend one sum of money at a particular time point has made it more difficult to find the necessary finance.

Two other factors relating to capital expenditure must also be considered, firstly the proliferation of manufacturers and secondly the increased work load of laboratories which has necessitated the purchase of additional instruments. By present day prices a small hospital department of clinical chemistry could well have a capital investment of up to £100,000 ignoring items of equipment costing less than £100. A medium sized laboratory might have an investment up to £250,000 and a large laboratory in excess of £400,000, again ignoring all items below £100. Amortization of these sums over a seven year period leads to the inescapable conclusion that there should be a significant financial input into these laboratories per annum. In practical terms this could mean that a laboratory would not receive any significant financial investment for two or three years followed by a single major outlay. Laboratory directors are then in a very different position if faced with a choice between two or three different instruments, provided by different manufacturers. An incorrect choice, that is incorrect on the basis of machine function, can leave the laboratory in a disadvantageous position for a period of up to five to seven years. Since a single disbursement of the order of £170,000 on a single item is not uncommon, extreme care must be exercised in the choice of instrument.

Major instrument manufacturers have now recognised the dilemma of the potential customer and reputable companies usually make arrangements either privately or through the appropriate health authority for some preliminary evaluatory work to be performed to provide information for customers to make a more informed choice. Based on practical experience, the position now seems to have been reached where knowledgeable laboratory directors will no longer entertain purchase of an instrument, even around £10,000, without recourse to some form of written report, or alternatively the opportunity for an in-depth appraisal of the instrument within their own laboratories. A number of documents are now in circulation dealing with various aspects of the evaluation of laboratory instruments (see appendix) and it is not the purpose of this article to further elaborate on these.

Over a period of years the author's laboratory has built up general and detailed experience in the evaluation of instruments ranging from hand held diluters to sophisticated multi channel analysers and in this paper some of the practical and organisational difficulties associated with equipment evaluation are reviewed. This may serve as a guide to other laboratories or persons who are contemplating undertaking such work.

Such factors range from relationships with manufacturers to insurance requirements and it is intended to deal with these areas on an item by item basis. Considerations are grouped in roughly three areas of priority and these will be indicated. Items which should be considered first are:

### Relationship with manufacturer

When performing an assessment of any instrument or piece of equipment the individual or laboratory concerned should attempt to develop a satisfactory relationship with the manufacturer, and this should be defined in writing. The conditions imposed on both sides will, to a large extent, be governed by how the initial contact is established. There are probably four ways in which this initial meeting arises.

*(i)* Where the manufacturer approaches the laboratory or individual and asks for advice or an evaluation.

*(ii)* Where the laboratory asks a manufacturer for permission to test and evaluate a loan instrument prior to a potential purchase.

*(iii)* Where the laboratory or individual asks a manufacturer for permission to test and evaluate a loan instrument and where any report is intended for distribution to public authorities, employers or other professional groups.

*(iv)* Where the laboratory or individual purchases an instrument either for routine use or with the intention of performing an evaluation.

It should be apparent that under sections *(i)* and *(iv)* the evaluating laboratory is essentially a free agent and is not beholden in any way to the manufacturer. In other words the manufacturer is not in a position to lay down conditions under which work is performed. Under *(iii)* certain constraints on the evaluation can be applied by the manufacturer and it is important at the outset to have a written agreement. Conditions applicable under *(ii)* are intermediary between the two aforementioned situations. Here the manufacturer is of course keen to obtain a potential purchase and consequently will be more receptive to conditions laid down by the laboratory.

### Publication of report

The conditions under which a written report would be available should be agreed, in writing, with the manufacturer. To some extent this would be dependent on how the initial liaison was developed but as a general recommendation laboratories should not undertake evaluatory work unless a written report can be made available to purchasing authorities or to professional groups. This comment does not apply where prototype equipment is tested. Consideration should be given at an early stage to the question of where the report is to be published or to be made available. Sometimes it is possible to publish such reports in the professional and technical press, but for very complex and large instruments the reports are so lengthy that in general they would not be acceptable to journal editors.

The first draft of any report should be immediately submitted to the manufacturer who would be free to add any addendum to the report to explain or justify any of the findings therein. This addendum should always be available in any final published report. It should be made clear at an early stage that no editing of the report by the manufacturer will be allowed, nor should the report be quoted out of context for advertising purposes.

### Production of report

Laboratories undertaking evaluations invariably underestimate the amount of time and effort required to produce a satisfactory report. When dealing with major pieces of equipment the report can often run to three hundred pages, including tables, graphs and figures and the time involved often approximates to that necessary to produce a PhD thesis. In particular the laboratory must have access to reserve clerical capacity for typing, photocopying, reference checking and other clerical duties. It is often found that the production of a report can take longer than the actual experimental evaluation itself.

### Costs involved in evaluation

These can be very significant for any type of evaluatory work and in particular those studies which are very labour intensive. Usually the major expenditure will be on staff time although this may not be the case where very expensive reagents and consumables are required. In the author's experience the cost of carrying out evaluations ranges from £100 to about £4,000 ignoring general overheads such as building and fabric costs. The major cost lies in the provision of experienced staff to perform the work and inevitably involves staff not directly concerned with the evaluation but also those in other parts of a laboratory. This comes about by the necessity, for example, to carry out comparative studies with similar instruments currently in routine use. The costing exercise usually performed when an evaluation is in progress relates to the costs of running the instrument and is quite distinct from the costs in carrying out an evaluation. In many cases the high costs of evaluation are not apparent because of the amalgamation or inclusion of the evaluatory procedure within the routine running of the laboratory. Eighty per cent of most costs of running a routine laboratory can be attributed to the provision of staff and as a rough guide any evaluator can simply add the number of man houvs used on the evaluation. In unusual circumstances additional costs may be incurred because of particular installation or consumable requirements. It is very important that laboratory directors be aware of the costs involved in evaluatory work when negotiating with instrument suppliers.

### Provision of consumables

This is partly related to costs but the point which must be made here is that a laboratory intending to undertake an evaluation should be very clear about what the strategy of the evaluation will be long before the instrument is installed. It is imperative that all the materials necessary to forward an evaluation and to complete it in as short a time as possible, should be at hand before the instrument arrives. The delay arising as a consequence of the necessity to order supplies during the course of an evaluation can be serious in terms of morale, and sometimes requires the work to be restarted from base line. In some cases specific materials are required and the supply of these should be negotiated with the manufacturer at an early date.

### Training

Before formally starting an evaluation it is extremely important that any operators should be fully trained in the use of the instrument. They should be trained by the manufacturer who should give notice in writing that he is satisfied with the technical competence and performance of the operator before the instrument is formally handed over for evaluation. It is necessary to stress this point since in the event of a poor technical report on the instrument the supplier must not be able to use the ability or the performance of the operating staff as an excuse for any unsatisfactory results. If possible training should take place on the manufacturer's premises, as well as allowing a period of familiarisation, after the installation, within the tester's laboratory. Training costs should be borne by the manufacturer.

The above remarks obviously only apply to complex instruments and the laboratory director must be guided in this area by examination of the capital cost and complexity of the instrument.

### Electrical evaluation

Arrangements should be made for a competent electrical or electronic engineer to examine the equipment for safety soon after installation. There are codes of practice relating to safety of electrically operated laboratory equipment (see appendix) and an examining engineer should be guided by these. In private industry the examiner may well come from within the organisation but public bodies often have recourse to government resources.

In the event of some unusual feature relating to electrical safety arising then any further evaluation should be suspended until the manufacturer has had a chance to comment.

The factors listed above are all of approximately equal importance in organising equipment evaluations. In the author's laboratory the following areas of interest are considered to be subsidiary to the headings listed above, but in the context of other laboratories they may take on a different order of priority.

### Staff

One fairly senior member of staff should be appointed as co-ordinator of the entire evaluation. He or she will also require one or more members of staff to work in a technical capacity and these persons should preferably be retained on this work throughout the evaluatory period. It is recommended that both the co-ordinator and one other member of staff should receive adequate training from the instrument supplier. Two members of staff should always be trained so that for long running evaluations no delays will occur as a result of sickness, holidays etc. If only one person can be trained then this should be the senior staff member, or co-ordinator. It is not wise to have a junior staff member trained who then passes on the necessary information to the more senior person. This often leads to errors in understanding and communication. The co-ordinator would be responsible for ensuring an adequate supply of the report. It is a serious mistake to have many different members of staff involved in such an evaluation as many errors and misconceptions can arise which impede the continuity of the project.

### Documentation

The co-ordinator in particular, should be familiar with, and have access to all standard documents relating to instrument testing and evaluation. These documents arise from many different sources depending on the type of work being undertaken, and any department undertaking such work should maintain a library of equipment testing procedures.

### Prototype instruments

When dealing with prototype equipment which is under development or manufacture, the commercial security of the maker should be safeguarded. The information obtained during testing then becomes confidential and circulation is restricted except by dispensation from the manufacturer. It is important to establish at the outset of any such work whether the instrument being tested falls into the prototype class or otherwise. Work for manufacturers on prototype instruments can usually attract a remuneration for the testing laboratory, but for marketable instruments, qualifications and examples as listed here should be in force.

### Periods of evaluation

A target date for the completion of a final report should be set in agreement with the manufacturer. This date will be conditioned to a large extent by the complexity of the instrument under test. This time period should not be underestimated; actual testing times, as opposed to report writing, can extend for up to six months, an equal period of time could well be apportioned to the production of a report. As a rough guide, a hand held pipette may take six working days to production of a report, an automatic diluter dispenser up to three weeks, a single channel continuous flow or discrete analyser up to six weeks and a fully automatic multi-channel analyser with associated computer facilities may take up to one year.

### Back-up facilities

By this is meant the ready access to laboratory computers or instruments using comparative methodologies. Analysis of results from testing procedures can usually be carried out using standard statistical packages available on most desk-top or mini computers. Occasionally it is necessary to formulate a statistical testing programme of an unusual nature, and it is then a distinct advantage to have an experienced computer programmer easily available, or better still, permanently working within the laboratory. On the question of comparative methodologies, many instrument testing procedures require that new instruments are measured against the analytical performance of machines which they are intended to replace. Not only does this tell the evaluator whether there has been a significant improvement in technology, and consequently in performance, but it also allows an examination of any defects in the methods which are being recommended by the manufacturer of the new instrument.

Since the evaluator is not only concerned with equipment performance but also with construction, reliability and safety, arrangements should be made at an early date to obtain professional advice and assistance from electronic engineers and physicists. Although the acceptance of an instrument is primarily conditioned by its analytical performance, the question of reliability and safety looms large in the requirements of a routine laboratory.

### Safety

Before carrying out any kind of analytical work the electrical safety of the instrument should first have been tested. Recommended standards are shown in the attached appendix.

Failure at this point necessitates returning the instrument to the manufacturer for modifications. Radiological and biological hazards to the operator must also be investigated. Biological hazards are not easy to establish and at the time of writing there are no formal testing procedures in print. The evaluator must be guided by the function of the instrument and the way in which it is constructed in order to arrive at a testing programme. A second feature relating to biological safety is that where service engineers are involved after an instrument has been adopted for routine use, their safety from biological hazards must be ensured. Practical sterilization procedures should be available before allowing service personnel to dismantle and adjust the instrument.

### Methods

At the outset of the evaluation it should be made clear that the instrument will be tested using methods recommended by the manufacturer and that modifications to methodologies will not be made during the course of an evaluation unless otherwise agreed. From the author's experience, it has been found that many manufacturers rely upon methodological developments being undertaken by the users. It is considered that a marketable instrument should be fully developed, both mechanically and analytically, before being sold. Agreement can be reached that a certain amount of method development will take place subsequent to a formal evaluation but this should be by defined agreement and should attract some form of remuneration to the testing laboratory.

The final points to be considered are possibly not quite so important as the aforementioned ones.

### Experience

Laboratories with no experience of evaluatory work are recommended to make a start in this field by only considering instruments of limited complexity. The purpose of this article is to draw attention to some of the problems and factors which must be considered before attempting work of a significant nature. Smaller laboratories are by no means precluded from indulging in this interesting and stimulating work but it is easy for them not to appreciate the extent of the work involved.

### Insurance

Before receiving an instrument for testing purposes its acceptance should first be cleared with any local administration. Some public and private authorities may disclaim responsibility for damage to, or caused by, an instrument which is not on the laboratory inventory. When a system is being tested prior to potential purchase within that establishment then it is likely that the local administration will raise no objections, but it is in the interests of both the manufacturer and the tester that this positon should be clarified at an early date.

### Servicing

When instruments are on extended loan, sometimes for periods of up to six months, arrangements should be made for the normal servicing to be performed and the testing schedule constrained to make allowances for this. These services should not be chargeable against the evaluating laboratory.

### Installation

Instrument requirements in the way of services, for example compressed air, vacuum, three phase electrical supply etc. should be noted and provided for, some time before accepting the instrument within the laboratory. This is a problem for the co-ordinator.

### Conclusion

It should be clear from the above that evaluations should not be undertaken lightly, that the laboratory performing such work has a responsibility both to its peer laboratories and to the instrument manufacturer, that a report be made available at an early date. Within the public industries it is important that such information be widely spread so that judicious choice of equipment can be made to provide public services at a cost which is realistic. It is strongly recommended that precis of reports, or the report themselves, should be available in the technical and professional press.

Evaluatory work is demanding, but can be enjoyable especially as one is testing instruments at the forefront of technology. By giving consideration to some of the points raised above, the evaluator will be guided round some of the problems which we have encountered in the last few years. Great care should be taken not to infringe the commercial or legal position of the instrument supplier and as long as the project is well documented from beginning to end then both parties are usually satisfied.

APPENDIX

The following publications are a source of further information.

1. Recommended scheme for the evaluation of instruments for automatic analysis in the clinical biochemistry laboratory. *Journal of Clinical Pathology,* **22,** page 278, (1969).
2. Ann. clin. Biochem. 11 (1974) 242. Technical Bulletin No. 33 Definitions of Some Words and Terms used in Automated Analysis.
3. Projet de Protocole D'Essai des Spectrophotometres. Doc. A-Version 4 - Decembre 1976. Societe Francaise de Biologie Clinique. Commission d'Essais des Appareils de Laboratoire.
4. Protocol for Establishing the Precision and Accuracy of Automated Analytic Systems. NCCLS Proposed Standard: PSEP-1 National Committee for Clinical Laboratory Standards.
5. Proposed Standard: PSI-3 Standard for Determining Spectrophotometer Performance Criteria. National Committee for Clinical Laboratory Standards.
6. Safety of Electrically operated Laboratory Equipment. Draft U.K. Document (Origin SIMA/BSI) for ultimate submission to IEC through SC62D-WG5.
7. Preparation of Manuals for Installation Operation and Repair of Laboratory Instruments. September 1972. National Committee for Clinical Laboratory Standards
8. Report to the Instrumentation Sub-Committee Assn. Clin. Biochem. The Assessment of the Cost of New Equipment. P. H. Lloyd August 1976.
9. NCCLS Proposed Standard: PSI-2. Standard for Temperature Calibration of Water Baths, Instruments and Temperature Sensors. NCCLS.
10. Proposed Standard: PSI-3 Standard for Determining Spectrophotometer Performance Criteria. NCCLS.
11. Guidelines for the Design of Laboratory Surveys. Laurence P. Skendsel, M.D. Am. Journal Clin. Path. Vol. 54. Munson Medical Center, Traverse City, Michigan 49684.

